# Tropoelastin: an *in vivo* imaging marker of dysfunctional matrix turnover during abdominal aortic dilation

**DOI:** 10.1093/cvr/cvz178

**Published:** 2019-07-08

**Authors:** Begoña Lavin, Sara Lacerda, Marcelo E. Andia, Silvia Lorrio, Robert Bakewell, Alberto Smith, Imran Rashid, René M. Botnar, Alkystis Phinikaridou

**Affiliations:** 1 School of Biomedical Engineering and Imaging Sciences, Department of Biomedical Engineering, King’s College London, 3rd Floor, Lambeth Wing, St Thomas’ Hospital, London SE1 7EH, UK; 2 Cardiovascular Division, BHF Centre of Excellence, King’s College London, London, UK; 3 Centre de Biophysique Moléculaire, CNRS, Orléans, France; 4 Radiology Department, School of Medicine, Pontificia Universidad Católica de Chile, Santiago, Chile; 5 Cardiovascular Division, Academic Department of Vascular Surgery, King’s College London, London, UK; 6 Wellcome Trust and EPSRC Medical Engineering Center, King’s College London, London, UK; 7 Pontificia Universidad Católica de Chile, Escuela de Ingeniería, Santiago, Chile

**Keywords:** Aneurysm, Dissections, Elastin, Tropoelastin, Matrix turnover, Molecular imaging, MRI

## Abstract

**Aims:**

Dysfunctional matrix turnover is present at sites of abdominal aortic aneurysm (AAA) and leads to the accumulation of monomeric tropoelastin rather than cross-linked elastin. We used a gadolinium-based tropoelastin-specific magnetic resonance contrast agent (Gd-TESMA) to test whether quantifying regional tropoelastin turnover correlates with aortic expansion in a murine model. The binding of Gd-TESMA to excised human AAA was also assessed.

**Methods and results:**

We utilized the angiotensin II (Ang II)-infused apolipoprotein E gene knockout (ApoE^-/-^) murine model of aortic dilation and performed *in vivo* imaging of tropoelastin by administering Gd-TESMA followed by late gadolinium enhancement (LGE) magnetic resonance imaging (MRI) and T_1_ mapping at 3 T, with subsequent *ex vivo* validation. In a cross-sectional study (*n* = 66; control = 11, infused = 55) we found that Gd-TESMA enhanced MRI was elevated and confined to dilated aortic segments (control: _LGE_=0.13 ± 0.04 mm^2^, control _R1_= 1.1 ± 0.05 s^-1^ vs. dilated _LGE_=1.0 ± 0.4 mm^2^, dilated _R1_ =2.4 ± 0.9 s^-1^) and was greater in segments with medium (8.0 ± 3.8 mm^3^) and large (10.4 ± 4.1 mm^3^) compared to small (3.6 ± 2.1 mm^3^) vessel volume. Furthermore, a proof-of-principle longitudinal study (*n* = 19) using Gd-TESMA enhanced MRI demonstrated a greater proportion of tropoelastin: elastin expression in dilating compared to non-dilating aortas, which correlated with the rate of aortic expansion. Treatment with pravastatin and aspirin (*n* = 10) did not reduce tropoelastin turnover (0.87 ± 0.3 mm^2^ vs. 1.0 ± 0.44 mm^2^) or aortic dilation (4.86 ± 2.44 mm^3^ vs. 4.0 ± 3.6 mm^3^). Importantly, Gd-TESMA-enhanced MRI identified accumulation of tropoelastin in excised human aneurysmal tissue (*n* = 4), which was confirmed histologically.

**Conclusion:**

Tropoelastin MRI identifies dysfunctional matrix remodelling that is specifically expressed in regions of aortic aneurysm or dissection and correlates with the development and rate of aortic expansion. Thus, it may provide an additive imaging marker to the serial assessment of luminal diameter for surveillance of patients at risk of or with established aortopathy.

## 1 Introduction

Abdominal aortic aneurysm (AAA) disease currently affects about 2% of men over 65 years of age.^1^ Aneurysmal segments can expand over time and have an elevated risk of rupture, which is associated with high mortality (~90%).^2^ Anatomical imaging for measurement of aortic diameter is currently used for the diagnosis and surveillance of AAA, where the risk of rupture correlates with aneurysmal size.^3^ However, as the majority of patients with AAA are asymptomatic, confirmed diagnoses often arise due to incidental findings or from dedicated ultrasound-based screening programmes in at-risk populations.^4,5^ Intervention using surgical or endovascular techniques is recommended when AAA diameters exceed 55 mm, expand rapidly (>10 mm/year) or in the context of symptomatic disease. However, predicting the progression and outcome of AAA remains complex due to the non-linearity of expansion rates^6^ and the absence of rupture or adverse events in a significant proportion (40%) of large aneurysms (>70–100 mm).^7^ Conversely, ≤20% of ruptured AAA is <55 mm in diameter. Thus, there is a currently, unmet clinical need to improve identification of high-risk patients and better inform subsequent management decisions. Molecular imaging strategies that characterize AAA composition and biology could improve existing risk stratification approaches that are solely derived from clinical and anatomical information.

Several pathogenic mechanisms have been implicated in aortic aneurysm formation and expansion including local inflammation,^8^ extracellular matrix (ECM) remodelling,^9–12^ increased elastolysis,^8^ and oxidative stress.^13^ Elastin is an abundant ECM protein of the aortic wall, the remodelling of which is crucial for the progression of AAAs.^9,10,12^ Elastin is formed by cross-linking of the 72 kDa soluble precursor tropoelastin molecule,^14^ by lysyl oxidase (LOX). Although it is well-known that active elastolysis leads to thinning and weakening of the elastic lamellae of the media during AAA formation,^9,10^ *de novo* synthesis of elastin fibres that also occurs has received little attention. Studies have shown that disordered elastogenesis is active in matrix remodelling in AAA^15^ and Marfan’s syndrome.^16,17^ However, newly synthesized tropoelastin monomers secreted from vascular smooth muscle cells ^18–20^ and macrophages^21^ frequently fail to cross-link into polymeric elastin fibres^12,21–23^ as a result of the reduced expression, absence or inactivation of LOX^24–29^ or any of the components of the microfibrillar scaffold required for fibre assembly.^30–32^

Because of their central role in the pathogenesis of AAA, *in vivo* imaging of aortic wall inflammation or matrix remodelling have been achieved using ultrasmall superparamagnetic particles of iron oxide (USPIOs),^33^ collagen-binding,^34^ and elastin-specific MRI contrast agents (Gd-ESMA).^35,36^ The clinical utility of USPIO-enhanced MRI was assessed in a *Phase II* study, in which imaging of vascular inflammation was shown to be predictive of aneurysmal growth and adverse events but did not provide incremental value beyond assessment of clinical risk factors.^33^ As dysfunctional matrix remodelling contributes to the reduction of vessel wall integrity and may promote aneurysm/dissection formation and rupture, we assessed the utility of a tropoelastin-specific magnetic resonance (MR) contrast agent in a murine model and excised tissue from human AAA. The results demonstrate that tropoelastin MRI leads to specific enhancement of aortic segments that develop aneurysm or dissection, which may improve risk assessment compared to the serial assessment of aortic diameter alone.

## 2 Methods

We utilized the Angiotensin II (Ang II)-infused ApoE^-/-^ murine model of aortic aneurysm and dissection, which shares some characteristics of the human disease including luminal dilation, macrophage infiltration, medial degradation, and thrombus.^11,12,37–39^ The term aortic dilation/expansion used in this article refers to both conventional aortic aneurysms and expanding aortic dissections as previously described to occur in this animal model.^12,39,41^ In a cross-sectional study, ApoE^-/-^ mice (*n* = 66) were imaged at a clinical 3 T MRI scanner before and at 1, 2, 3, and 4 weeks after infusion of Ang II (*n* = 8/per group). Magnetic resonance angiography (MRA), late gadolinium enhancement (LGE), and T_1_-mapping images were acquired. Ang II-infused mice treated with pravastatin and aspirin (*n* = 10) were scanned at 4 weeks. In a longitudinal study (*n* = 19), Ang II-infused mice were scanned weekly for up to 4 weeks. Excised murine aortas were collected for histology (*n* = 4), western blotting (*n* = 3), and inductively coupled plasma mass spectrometry (*n* = 4) per time point. Excised human aortic aneurysm tissues (*n* = 4) were scanned and analysed *ex vivo*. The tropoelastin and elastin-binding agents were administered intravenously on consecutive days (0.2 mmol/kg). Numeric data were analysed for normality and the significance was determined using the appropriate non-parametric test. Box–whisker plots were used in the figures, whereas the numeric data presented in the abstract are mean ± standard deviation. Animal procedures and use of human samples were approved by the appropriate committees. Detailed methods and statistics are described in the Supplementary material online, Figure S1.

## 3 Results

*In vivo* MRA showing examples of aortic dilation with *ex vivo* validation and a summary of Ang II-induced aortic aneurysms and dissections observed in our study are demonstrated in the Supplementary material online, Figure S2.

### 3.1 Gd-TESMA MRI demonstrates elevated tropoelastin expression confined to dilated aortic segments

**Figure 1:**
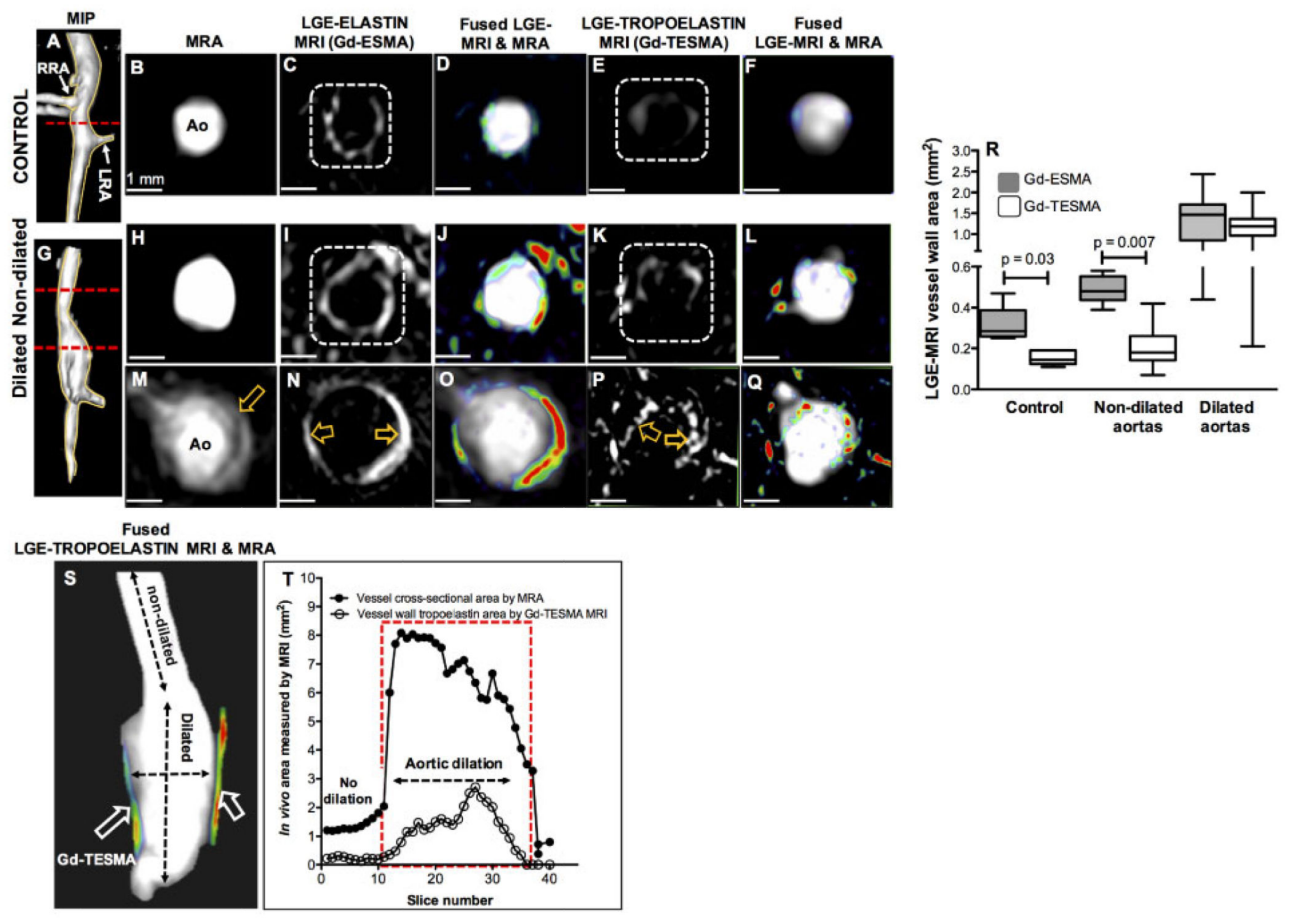
Gd-TESMA MRI demonstrates elevated tropoelastin expression confined to dilated aortic segments. (*A*–*Q*) MRA and LGE-MRI of a control (*A*–*F*) and an Ang II-infused ApoE^-/-^ mouse with aortic dilation (*G*–*Q*) scanned with the elastin (*B–D*, *H–J*, and *M–O*) and 24 h later the tropoelastin contrast agents (*E* and *F*; *K* and *L*; and *P* and *Q*). (*R*). Box–whisker plots of the LGE-MRI area shows that, unlike Gd-ESMA, the uptake of Gd-TESMA is specific for dilated segments (*n* = 14) of the aorta, consistent with dysfunctional matrix turnover in these regions, and is low in control (*n* = 5) and Ang II-infused mice without dilation (*n* = 6). Quantitative data were analysed by a Wilcoxon signed rank test for paired samples. (*S* and *T*) Fusion of reformatted MRA and LGE-MRI images from an Ang II-infused ApoE^-/-^ mouse, after administration of Gd-TESMA, and graph show that the uptake of the tropoelastin contrast agent is confined within the dilated wall. Ao, aorta; LRA: left renal artery; MIP, maximum intensity projection; RRA, right renal artery.

As elastin is abundant throughout normal and dilated aortic segments, we performed imaging using the elastin (Gd-ESMA) and tropoelastin (Gd-TESMA) specific MR agents in control and Ang II-infused ApoE^-/-^ mice (24 h apart) to compare total elastin and tropoelastin expression within the aortic wall, given that Gd-ESMA binds equally to both cross-linked elastin and tropoelastin.^35^ As previously demonstrated,^40^ Gd-ESMA resulted in circumferential enhancement of the control aortic wall consistent with binding to cross-linked elastin, which is an integral structural component of the normal aorta (Figure 1A–D). Conversely, there was little enhancement of control vessel wall after injection of Gd-TESMA, demonstrating that tropoelastin expression is not present or up-regulated in the absence of disease (Figure 1E and F), which was verified histologically and as previously shown.^40^ In an Ang II-infused ApoE^−/−^ mouse (Figure 1G), LGE-MRI through a non-dilated segment of the aorta showed circumferential enhancement with Gd-ESMA (Figure 1H–J) and little enhancement with Gd-TESMA (Figure 1K and L). Conversely, LGE-MRI through a dilated segment of the aorta showed uptake of both agents (Figure 1M–Q). Comparatively, Gd-ESMA enhancement resulted in a strong circumferential enhancement of the dilation (Figure 1M–O), whereas Gd-TESMA of enhancement was patchy (Figure 1P and Q). Quantitatively, whilst the Gd-ESMA enhanced area was larger compared to that of Gd-TESMA in control mice and animals infused with Ang II but did not develop aortic dilations, similar areas of enhancement were measured in dilated aortic segments for both contrast agents (Figure 1R). Fused three-dimensional reformatted MRA and Gd-TESMA enhanced images together with a graph show the changes in aortic and vessel wall enhanced areas from consecutive slices along the aorta in another Ang II-infused mouse (Figure 1S and T). A comparison of the quantitative differences between Gd-ESMA and Gd-TESMA are illustrated in Supplementary material online, Figure S3. These data demonstrate that unlike Gd-ESMA, the uptake of Gd-TESMA is specific for dilated segments of the aorta, consistent with dysfunctional matrix turnover in these regions.

### 3.2 MRI of tropoelastin shows enhancement at sites of aortic aneurysm or dissection

**Figure 2:**
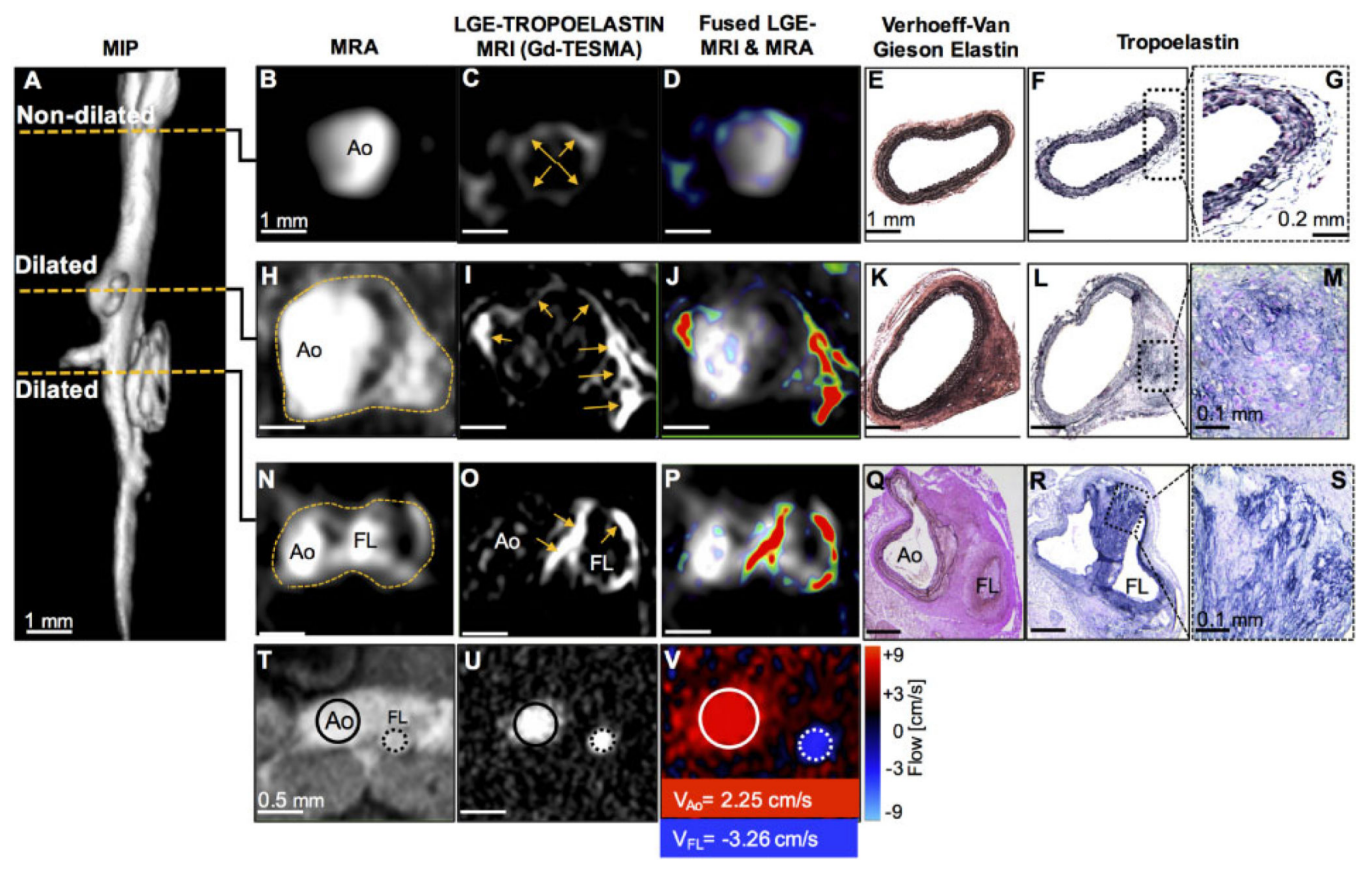
MRI of tropoelastin shows enhancement at sites of aortic aneurysm or dissection. (*A*) A reformatted MRA of an Ang II-infused ApoE^-/-^ mouse shows two regions of aortic dilation. (*B*–*G*) MRA, LGE-MRI, and histology of a non-dilated segment show a normal aortic size, low enhancement after administration of Gd-TESMA, and lack of tropoelastin, respectively. (*H*–*M*) At the level of the first aortic dilation, there is vascular enhancement of the aneurysm after administration of Gd-TESMA that co-localized with the accumulation of tropoelastin as verified histologically. (*N*–*P* and *T*–*V*) At the level of the second aortic dilation, MRI images show the formation of a false lumen indicative of an aortic dissection, with aortic enhancement after administration of Gd-TESMA and retrograde blood flow. (*Q*–*S*) Histology verified the formation of two lumens and the accumulation of tropoelastin in areas where vascular enhancement was observed *in vivo* using the tropoelastin agent. Ao, aorta; FL, false lumen.

A three-dimensional reformatted angiogram acquired from an Ang II-infused ApoE^−/−^ mouse for 2 weeks showed a non-dilated segment of the aorta and two separate regions of aortic dilation (Figure 2A). MRI slices acquired through the non-dilated segment of the aorta showed a normal aortic diameter (Figure 2B) and weak Gd-TESMA enhancement (Figure 2C and D). This was verified histologically by the absence of aortic dilation (Figure 2E) and lack of tropoelastin monomers (Figure 2F and G).

In a distal segment (suprarenal), angiography showed a region of aortic aneurysm (Figure 2H) and vessel wall Gd-TESMA enhancement (Figure 2I and J) that was validated histologically by the deposition of tropoelastin within the remodelled media (Figure 2K–M). At the second level of aortic dilation, angiography and quantitative flow images showed formation of a false lumen (Figure 2N) with retrograde blood flow (Figure 2T–V) indicative of an expanding aortic dissection with a medial tear, as previously described for this model.^39,41^ In this case, Gd-TESMA enhancement was observed in the vessel wall between the true and false lumens, with circumferential enhancement of the false lumen margins (Figure 2O–P). The presence of dual (true and false) lumens was confirmed by histology (Figure 2Q), with abundant tropoelastin (Figure 2R and S) corresponding to areas of Gd-TESMA enhancement seen on *in vivo* MRI.

### 3.3 Quantitative MRI and tissue analysis shows that tropoelastin accumulation is confined within dilated aortic segments

**Figure 3:**
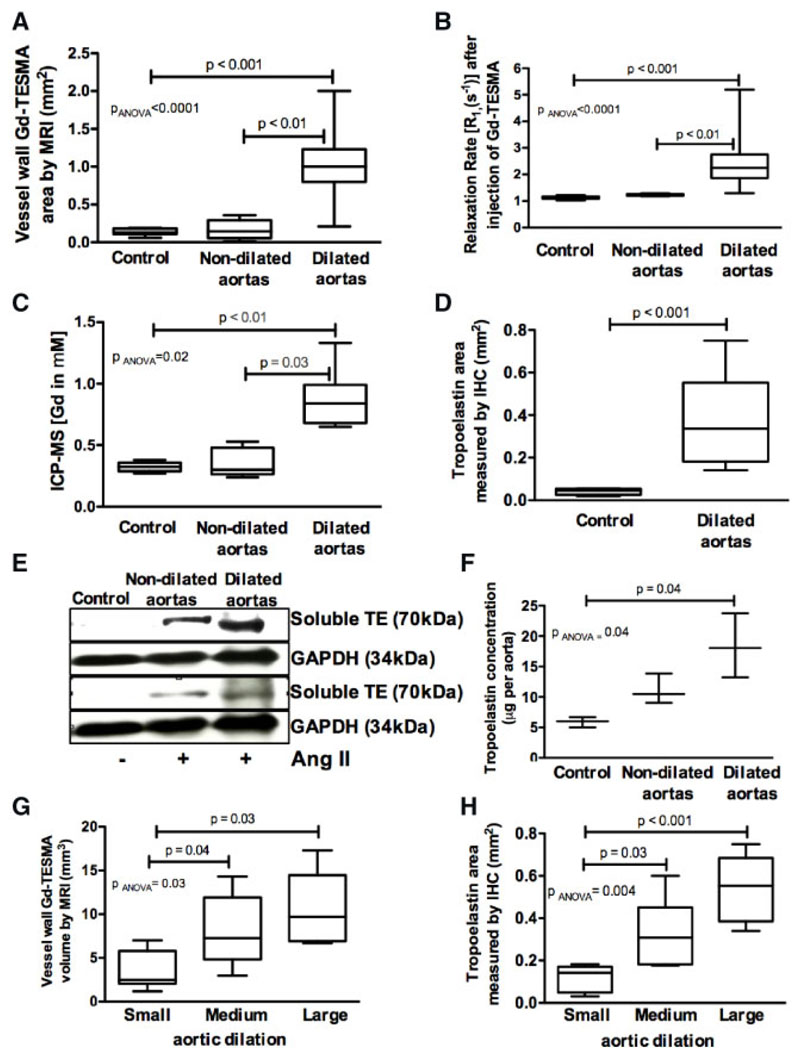
Quantitative MRI and *ex vivo* tissue results show that tropoelastin accumulates within dilated aortas. (*A* and *B*) Quantitative Gd-TESMA LGE-MRI measurements showed larger enhancement area and higher vessel wall R_1_ in dilated (*n* = 36), compared to non-dilated (*n* = 6) and control vessels (*n* = 6). (*C*–*F*) ICP-MS (*n* = 4/group), immunohistochemistry (*n* = 4 for control, *n* = 17 for dilated), and western blotting (*n* = 3/group) verified the *in vivo* MRI findings and showed increased gadolinium concentration and tropoelastin accumulation in dilated compare to non-dilated and control aortas. (*G* and *H*) Tropoelastin accumulation was higher in medium and large-size compared to small-size dilation as measured by both *in vivo* MRI (*n* = 12/group) and immunohistochemistry (*n* = 6/group). Two groups were compared with a Mann–Whitney test and multiple groups with a Kruskal–Wallis ANOVA test followed by a Dunn’s *post hoc* test.

Quantitative MRI measurements showed larger Gd-TESMA enhanced area and higher vessel wall R_1_ in dilated, compared to non-dilated and control aortas (Figure 3A and B)*. Ex vivo* ICP-MS, immunohistochemistry, and western blotting showed increased gadolinium concentration and tropoelastin accumulation in dilated compared to non-dilated and control aortas (Figure 3C–F). Tropoelastin deposition was higher in medium and large-size compared to small-size dilated aortic segments as measured by both *in vivo* Gd-TESMA enhanced MRI and immunohistochemistry (Figure 3G–H). Correlation analyses showed good agreement between MRI and histological measurements (Supplementary material online, Figure S4). Quantification of soluble tropoelastin in serum showed no differences between controls, untreated, and treated Ang II-infused mice with dilated aortas (Supplementary material online, Figure S5).

### 3.4 Longitudinal MRI of tropoelastin shows that tropoelastin accumulation correlates with aortic expansion

**Figure 4:**
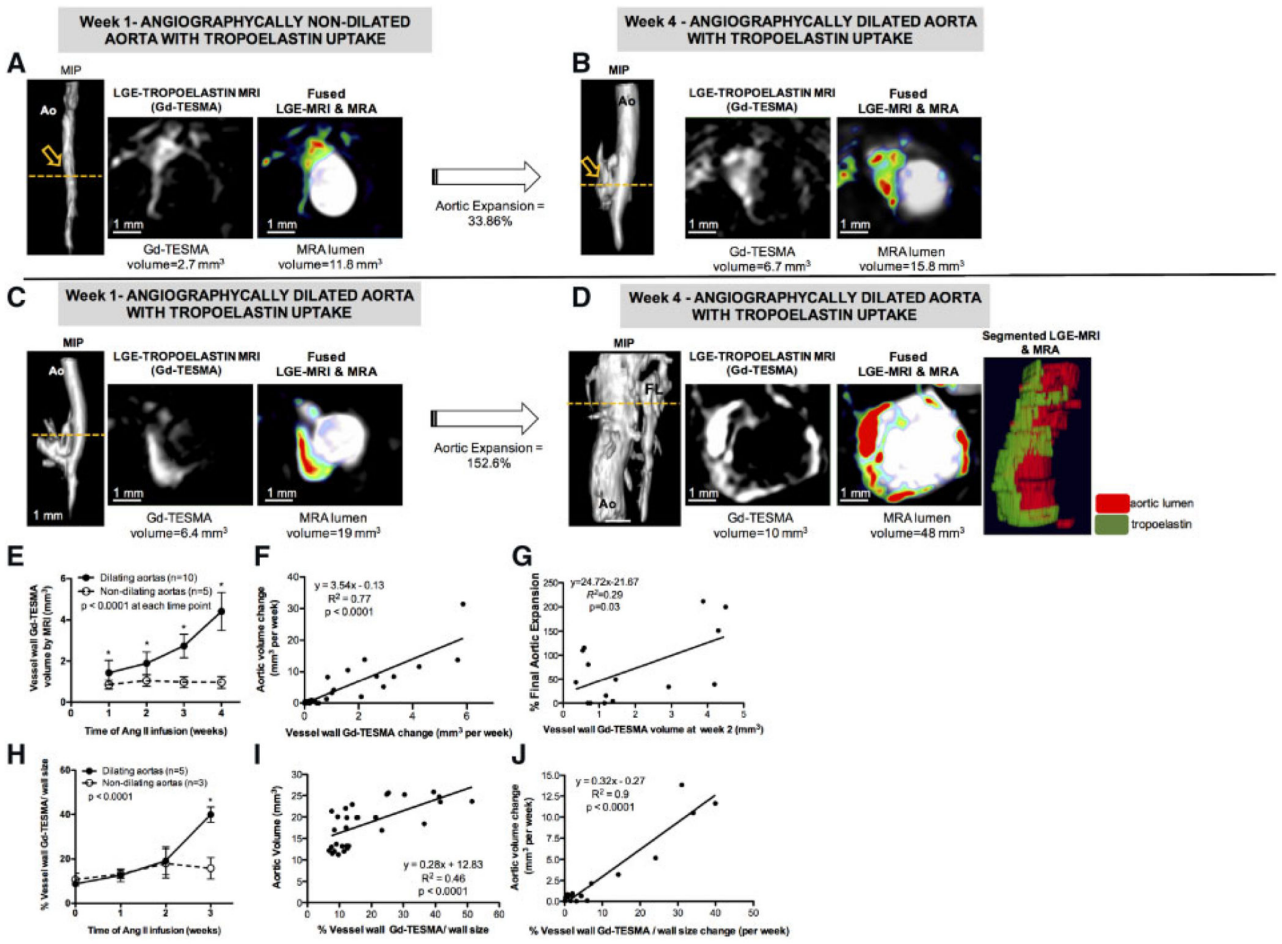
Longitudinal MRI of tropoelastin shows that tropoelastin accumulation correlates with aortic expansion. (*A* and *B*) MRA and Gd-TESMA enhanced images, show enhancement in a non-dilated aorta at Week 1 following Ang II-infusion, which subsequently dilates at Week 4. (*C* and *D*) MRA and Gd-TESMA enhanced images, show a higher enhancement in an already dilated aorta at Week 1 following Ang II-infusion, which further dilates and accumulates more tropoelastin at Week 4. (*E*) Dilating aortas contained more tropoelastin compared to non-dilating aortas at all times. (*F*) The rate of aortic expansion correlates with the rate of tropoelastin deposition. (*G*) The Gd-TESMA enhanced area measured at 2 weeks after Ang II-infusion correlated with aortic expansion measured at 4 weeks (*E* and *G*, *n* = 15). (*H*) Dilating aortas contain higher proportion of tropoelastin at Week 3 following Ang II infusion. (*I*) The proportion of tropoelastin in the vessel wall correlates with aortic volume. (*J*) The increase in the proportion of tropoelastin correlates with the rate of aortic expansion (*H–J*, *n* = 8). Two groups were compared with a Mann–Whitney test and multiple groups were compared with a Kruskal–Wallis followed by a Dunn’s *post hoc* test. Ao, aorta; MIP, maximum intensity projection; FL, false lumen.

Next, we performed a proof-of-concept longitudinal study, in which 19 Ang II-infused ApoE^-/-^ mice were scanned weekly for up to 4 weeks to investigate the effect of tropoelastin accumulation on the rate of aortic expansion and incidence of rupture. One mouse died because of a ruptured aorta 8 days after infusion and three mice died from undetermined reasons. Of the 15 remaining mice, 10 had dilating and five had non-dilating aortas. An example of an animal with increased Gd-TESMA enhancement in a non-dilated aorta, which subsequently enlarged is shown in Figure 4A and B. This animal had an angiographically normal size aorta and Gd-TESMA enhancement 1 week after Ang II-infusion (Figure 4A). Subsequently, at Week 4 there was a 33.86% dilation of the aorta and further increase in Gd-TESMA enhancement (Figure 4B). An example of an animal with increased Gd-TESMA enhancement in a dilated aorta which further enlarged by Week 4 is shown in Figure 4C and D. This animal had an angiographically dilated aorta and higher Gd-TESMA enhancement 1 week after Ang II-infusion (Figure 4C). At Week 4, there was a 152.6% expansion of the aorta and formation of a false lumen. Importantly, Gd-TESMA enhanced images showed a significant increase in tropoelastin accumulation around the aortic dilation. The segmented tropoelastin was super-imposed on the segmented aortic lumen as seen by Gd-TESMA MRI and MRA, respectively. Quantitatively, dilating aortas had higher Gd-TESMA enhancement compared with non-dilating aortas at all-time points (Figure 4E). The rate of aortic expansion, as measured using MRA, correlated with the rate of tropoelastin accumulation, as measured by Gd-TESMA enhancement (Figure 4F). The Gd-TESMA enhancement measured at 2 weeks after Ang II-infusion correlated with the final aortic expansion (Figure 4G). When the volume of Gd-TESMA enhancement was normalized to vessel wall size, we found a higher proportion of Gd-TESMA in dilating compared to non-dilating aortas 3 weeks after Ang II-infusion (Figure 4H). The proportion of Gd-TESMA correlated with aortic size (Figure 4I) and the rate of aortic expansion (Figure 4J).

### 3.5 Pravastatin and aspirin treatment did not reduce tropoelastin accumulation or aortic dilation

**Figure 5:**
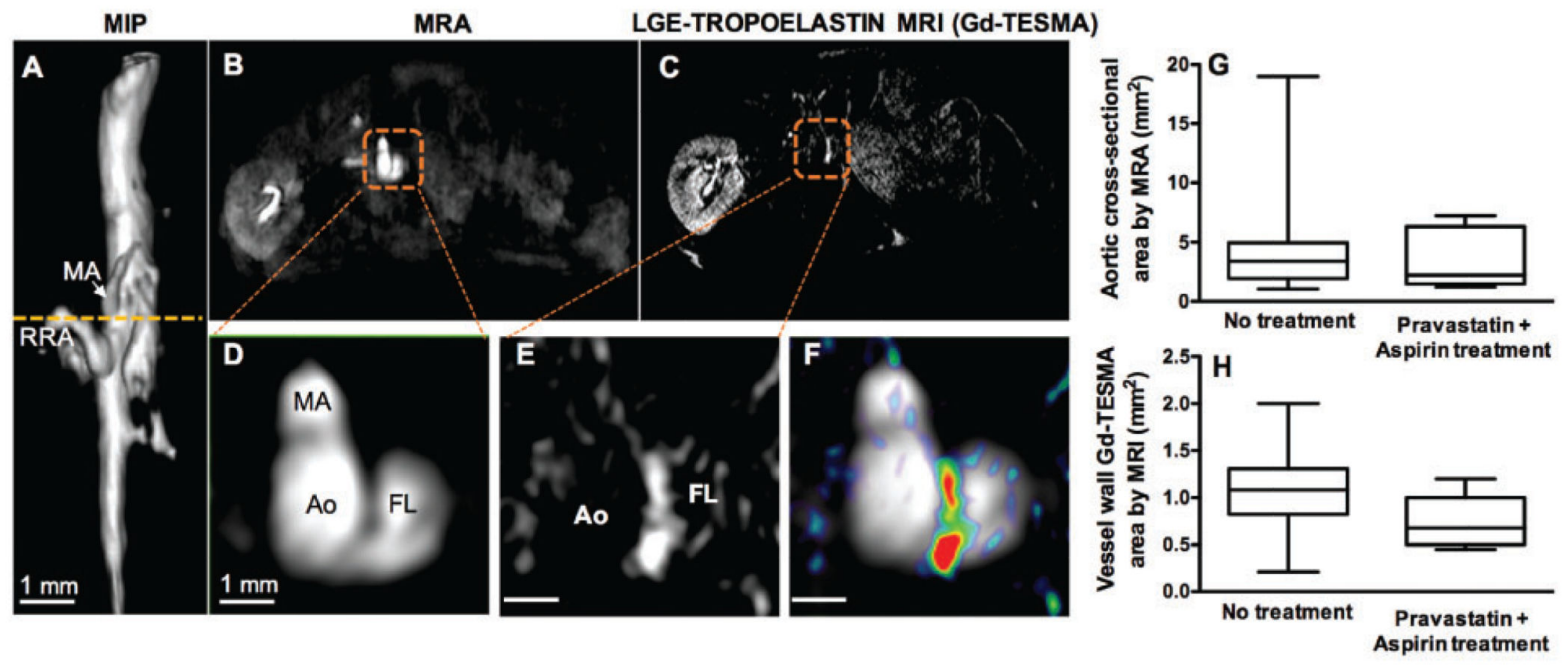
Concurrent treatment with pravastatin and aspirin did not reduce tropoelastin accumulation or aortic dilation. (*A*) A reformatted angiogram acquired from an Ang II-infused ApoE^-/-^ mouse treated with pravastatin and aspirin shows a suprarenal dilation. (*B*–*F*) Cross-sectional MRA and Gd-TESMA enhanced images, showed enhancement of the aortic dissection. (*G* and *H*) Quantification of the aortic cross-sectional and Gd-TESMA enhanced areas were similar between treated (*n* = 10) and untreated mice (*n* = 36). Quantitative data were compared with a non-parametric Mann–Whitney test. Ao, aorta; FL, false lumen; MA, mesenteric artery; MIP, maximum intensity projection; RRA, right renal artery.

We then tested whether quantifying tropoelastin turnover could be used as an imaging marker to assess treatment response. As aspirin and statins reduce clinical events and are commonly prescribed together in patients with established coronary artery disease, we assessed whether administration of these therapies reduces tropoelastin expression in this model.^42,43^ Therefore, we treated a subgroup of Ang II-infused mice with pravastatin and aspirin for 30 days. In our study, seven out of the 10 (70%) treated mice developed aortic dilation, which was similar to the rate observed for untreated animals. MRI images of a treated mouse are shown in Figure 5. The three-dimensional reformatted angiogram shows the aortic dilation (Figure 5A) and the transverse images reveal a medial dissection and a false lumen (Figure 5B–D). Gd-TESMA enhancement occurred in the vessel wall between the true and false lumens (Figure 5E and F). *In vivo* MRI showed no differences in the aortic cross-sectional and Gd-TESMA enhanced areas between treated and untreated animals (Figure 5G and H).

### 3.6 Gd-TESMA MRI identifies tropoelastin accumulation in human aortic aneurysms

**Figure 6:**
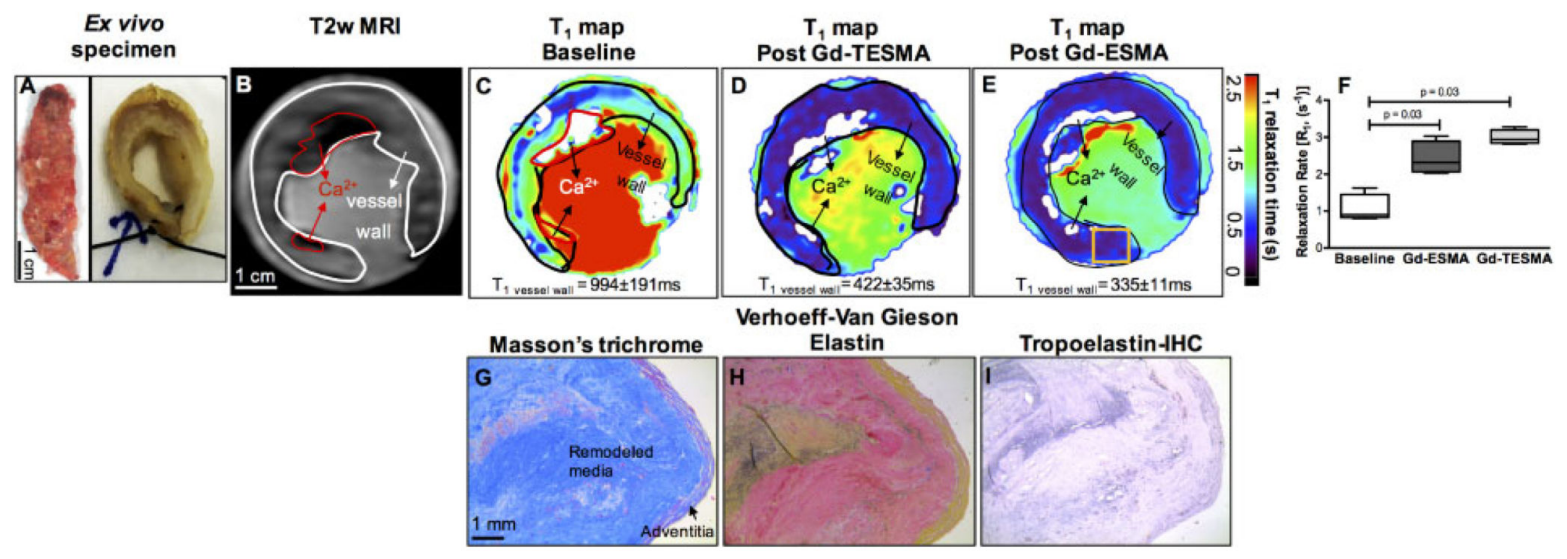
Gd-TESMA MRI identifies tropoelastin accumulation in human aortic aneurysms. (*A*) *Ex vivo* photograph of an aortic aneurysm in longitudinal and transverse planes after suturing the ends for the imaging experiment. (*B* and *C*) A T2W image shows the remodelled vessel wall and a corresponding T_1_ map shows the baseline relaxation times. (*D*) A T_1_ map acquired after soaking the specimen in Gd-TESMA followed by washing in buffered saline to eliminate unspecific binding shows uptake of the agent and reduction of the T_1_ relaxation time. (*E*) A repeated T_1_ mapping experiment after washing the specimen, soaking it in Gd-ESMA, followed by another wash also showed a reduction of the T_1_ values. (*F*) Quantification of the relaxation rate (R_1_) changes shows significant uptake of both agents in the aneurysmal wall. Quantitative data were analysed by a Wilcoxon signed rank test for paired samples (*n* = 4). (*G*–*I*) Corresponding histology shows the tissue morphology and deposition of tropoelastin within the vessel wall.

To test the translational potential of our work, we imaged tissue obtained from patients with AAA with the tropoelastin (Gd-TESMA) and elastin (Gd-ESMA) contrast agents. Excised AAA (Figure 6A) was imaged with a T2W scan (Figure 6B) to identify the vessel wall. Subsequent, T_1_ mapping experiments before (Figure 6C) and after soaking the tissue showed retention of both agents in the aneurysmal vessel wall (Figure 6D–F) and *ex vivo* immunohistochemistry verified the accumulation of tropoelastin in the media (Figure 6G–I).

## 4 Discussion

We investigated the role of tropoelastin as a new imaging marker of dysfunctional matrix turnover in aortic aneurysms and dissections in the Ang II-infused ApoE^-/-^ murine model and excised human aortic aneurysmal tissue. *In vivo* imaging of tropoelastin was performed using a gadolinium-based tropoelastin-specific magnetic resonance contrast agent (Gd-TESMA). Gd-TESMA enhanced MRI showed that tropoelastin accumulation is elevated and confined to dilated aortic segments and identifies aortic dilation with different morphological characteristics. Gd-TESMA enhanced MRI also showed that the proportion of tropoelastin in the vessel wall is higher in dilating compared to non-dilating aortas and correlates with the rate of aortic expansion. Pravastatin and aspirin treatment did not reduce the tropoelastin turnover or aortic dilation in this animal model, possibly reflecting the lack of consistent evidence regarding their efficacy for management of AAA in clinical studies. Importantly, Gd-TESMA enhancement demonstrated accumulation of tropoelastin in excised human aneurysms, highlighting the translational potential of this imaging strategy. Our results suggest that tropoelastin MRI is a novel approach for the identification of dysfunctional matrix remodelling in aortic disease, which correlates with the development and rate of aortic dilatation.

Our cross-sectional study and quantitative analysis in mice showed that the uptake of Gd-TESMA was confined to and higher in dilated compared with non-dilated and control aortic segments and it was also higher in aortic tissue from medium and large compared to small-dilated aortic segments, suggesting that tropoelastin is a disease-specific marker that correlates with the degree of aortic expansion. Importantly, *in vivo* monitoring of vascular tropoelastin remodelling was detectable and feasible at clinical field strengths. *In vivo* enhancement of the dilated aortic segments and higher vascular R_1_ values were corroborated by the accumulation of tropoelastin, as detected by *ex vivo* immunohistochemistry and western blotting, and by higher gadolinium concentration as measured by inductively coupled plasma mass spectrometry. Consistent with our results, the detrimental effects of increased elastolysis, oxidative stress, and impaired elastin cross-linking in promoting aortic dilation have also been previously reported in the Ang II-infused, the elastase-perfused, the fibrillin-1 knock-out/Marfan syndrome, and the LOX knock-out murine models.^11–13,29,37,44–47^

Our results using excised human aortic aneurysms demonstrate that the tropoelastin contrast agent maintains its specificity towards human tropoelastin and can be detected using a clinical scanner, highlighting the translational potential of our work. As dissected human thoracic aneurysms are characterized by elevated elastin content with reduced insoluble elastin indicative of ineffective maturation and cross-linking, there is evidence for an imbalance between elastin synthesis and degradation in effected aortic segments that could potentially be identified using a Gd-TESMA MRI approach.^21–23,48^ This could be extended to patients with genetic disorders of the ECM, such as cutis laxa and Marfan syndrome, where altered elastin homoeostasis has also been identified.^17,49^ As macrophages have been shown to be a source of tropoelastin, there is a possible link between aortic inflammation and disordered elastin synthesis in the pathogenesis of AAA,^21^ which may also be present in atherosclerosis effecting small and medium-sized arteries. The current study expands on previous work that assessed total elastin content, using Gd-ESMA, in models of AAA^35,36^ through the development and application of a probe (Gd-TESMA) that specifically targets tropoelastin in order to identify areas of pathological elastin turnover that are only present in diseased vessels.^40^ Here, we found that uptake of Gd-TESMA is confined within dilated walls and, unlike Gd-ESMA, it eliminates signal from endogenously present cross-linked elastin that exists in both control and diseased vessels.

Our proof-of-principle longitudinal study in mice showed that the proportion of tropoelastin is higher in dilating aortas and correlated with the rate of aortic expansion. This finding has potential implications for the screening or surveillance of aortic aneurysms and dissections with non-invasive imaging. Currently, population screening of high-risk patients including males, greater than 65 years of age with a history of smoking has been established in some countries and is associated with halving the mortality rate associated with AAA.^4,5^ However, continued surveillance of AAA and prediction of expansion is challenging because of the unpredictability and non-linearity of expansion rates^6^ despite the development of reliable angiography techniques^50^ and standardized reporting systems that offer excellent inter-observer agreement in assessing vascular abnormalities. Addition of an imaging marker, such as the tropoelastin, that can be quantified non-invasively and which correlates with aortic expansion might have additive value in patient risk stratification. This may be particularly important for patients in whom treatment decisions are not straightforward, including patients with aneurysms that are borderline for intervention (50–55 mm). Furthermore, we observed elevated tropoelastin levels in both areas of aneurysmal dilation and aortic dissection, likely indicating common pathophysiological links between these conditions that could potentially be identified using a common imaging strategy with Gd-TESMA MRI. As patients with underlying connective tissue disease have an increased incidence of both aortic aneurysm formation and aortic dissection, molecular imaging of tropoelastin may be informative for both conditions that appear to be driven by dysfunctional matrix remodelling.

Currently, there is no proven specific therapy that reduces aneurysm growth.^51^ For this reason, we chose to administer a medication regimen comprising of commonly prescribed therapies for patients with atherosclerotic vascular disease (i.e. aspirin and statins) to assess whether any modulation of dysfunctional matrix turnover within the aortic aneurysm and dissections could be observed using Gd-TESMA enhanced MRI. We found that concurrent treatment with pravastatin and aspirin did not reduce tropoelastin accumulation, aneurysm formation or aortic dissection in Ang II-infused ApoE^-/-^ mice. Despite the unequivocal benefits of statins in reducing the rate of plaque progression animal models^52^ and cardiovascular events in patients with coronary artery disease,^53^ there are conflicting data regarding the effect of statins in reducing aneurysm growth and incidence of rupture, indicative of important aetiological differences underlying these two vascular diseases.^54^ Similarly, statin-treatment in Ang II-infused mice has yielded conflicting results.^55,56^ Preservation of medial elastin lamellae via inhibition of matrix metalloproteinases and reduction of aortic diameter was reported only when pre-activated simvastatin was administered subcutaneously,^55^ instead of orally for the same duration, in Ang II-infused mice as reported here and in previous studies.^56^ As standard therapies for atherosclerosis have yielded mixed results in the context of aortic disease, there is a need to identify novel therapeutic targets such as the inhibition of c-Jun N-terminal kinase^45^ and miR-29b (microRNA)^57^ that showed aneurysm reduction via modulation of ECM metabolism. In light of these results, molecular imaging of tropoelastin could provide a surrogate marker for testing the efficacy of novel therapies in translational studies.

The current study has some limitations. Firstly, although tropoelastin expression was associated with aortic aneurysms and dissections, aortic rupture with intra-abdominal haemorrhage did not occur in a sufficient number of animals and thus, we were unable to test the value of imaging tropoelastin in predicting vessel wall instability. Secondly, region-of-interest analysis was performed manually because of the lack of an automated/semi-automated software that could potentially decrease user bias. However, there was a good inter-observer agreement for the analysis of MRI data.

We show, for the first time, that molecular MRI using a tropoelastin contrast agent at clinical field strengths can non-invasively quantify dysfunctional matrix remodelling, which was specifically observed at sites of aortic aneurysm and dissection. Importantly, longitudinal assessment demonstrated that the proportion of aortic tropoelastin expression correlated with the rate of aortic expansion that could have potential implications for screening and risk stratification of patients. Although, the importance of disordered elastin turnover in aortic aneurysm/dissection is well established,^21–23,48^ *in vivo* detection of dysfunctional elastin remodelling has been hampered by the lack of a clinically validated, non-invasive diagnostic tool. An imaging strategy using the tropoelastin-specific MR contrast agent has several potential advantages. Firstly, as the probe does not bind to endogenous cross-linked elastin present in the normal aortic wall, it allows for specific detection of areas of dysfunctional matrix remodelling in active aortic vessel wall disease. Secondly, tropoelastin MRI could allow for the direct localization and quantification of the effect of treatments that aim at modulating tropoelastin turnover. Thirdly, the use of T_1_ maps and imaging at a clinical field strength makes our approach quantitative and clinically translatable. Finally, the versatility of the probe to be chelated with radioisotopes makes it also suitable for nuclear/hybrid imaging, further increasing its translational potential.

In conclusion, tropoelastin MRI identifies dysfunctional matrix remodelling that is specifically expressed in regions of aortic aneurysm or dissection and correlates with the development and rate of aortic expansion. Thus, it may provide an additive imaging marker to the serial assessment of luminal diameter for surveillance of patients at risk of or with established aortopathy.

## Supplementary Material

Supplementary material is available at *Cardiovascular Research* online.

Supplementary Data

## Translational perspective

Abdominal aortic aneurysms and dissections are characterized by dysfunctional matrix remodelling, with accumulation of monomeric tropoelastin rather than cross-linked elastin that may contribute to progressive dilation and rupture. Using a tropoelastin-specific magnetic resonance imaging (MRI) contrast agent (Gd-TESMA), we demonstrate that aortic accumulation of tropoelastin is confined to regions of aortic dilation and that the proportion of tropoelastin accumulation correlates with the rate of aortic expansion in a murine model of aortic dilation. As Gd-TESMA enhanced MRI identified tropoelastin accumulation in human aneurysmal tissue, this approach may provide an imaging marker of disease activity and vessel wall integrity additional to serial measurement of aortic diameter.
