# Supplementary material for: Tropoelastin: an in vivo imaging marker of dysfunctional matrix turnover during abdominal aortic dilation
Source: Cardiovasc Res. Author manuscript; Available in PMC 2020 Apr 1. (PMC7104357; doi:10.1093/cvr/cvz178)
Supplement: Supplementary Data [file EMS84567-supplement-Supplementary_Data.docx]

**Supplementary material online**

**TITTLE: Tropoelastin: An *in vivo* imaging marker of dysfunctional matrix turnover during abdominal aortic dilation**

**AUTHORS:** Begoña Lavin, Sara Lacerda, Marcelo E. Andia, Silvia Lorrio, Robert Bakewell, Alberto Smith, Imran Rashid, René M. Botnar and Alkystis Phinikaridou

**APPENDIX**

**Supplementary Methods**

**Tropoelastin-binding and elastin-binding probes:** The tropoelastin-binding MR contrast-agent (Gd-TESMA; 1984.16 Da) was synthesized and prepared as we have previously described ^1^. The elastin-specific contrast agent complexed with gadolinium (Gd-ESMA; 856 Da) was provided by Lantheus Medical Imaging (North Billerica, USA).

**Murine model.** The Angiotensin II (Ang II)-infused murine model of aortic aneurysm and dissection was used as previously described ^2-5^. Homozygous male ApoE^-/-^ mice (B6.129P2–ApoE^tm1Unc^/J) were purchased from Charles Rivers Laboratories (Edinburgh, UK). 8-10 week-old, male, ApoE^-/-^ mice were implanted with mini-osmotic pumps (Alzet model 2004, Durect Corp, Cupertino, CA, USA) that released angiotensin-II subcutaneously at a dose of 1000 ng kg^-1^min^-1 2-4, 6^. Gender-matched ApoE^-/-^ not infused with Ang II were used as controls (n=11). In a cross-sectional study ApoE^-/-^ mice (n=55) were implanted with mini-osmotic pumps and imaged before and at 1, 2, 3 and 4 weeks after Ang II infusion (n=8/per group). In the treatment group, ApoE^-/-^ mice (n=10) received pravastatin (40mg/kg/day) (Kemprotec Ltd, Middlesborough, UK) and aspirin (100 mg/kg/day) administered in the drinking water simultaneously with Ang II infusion for 4 weeks. Following the MRI scans, the animals were culled with a ketamine/xylazine overdose and tissues were harvested for *ex vivo* analyses. In a longitudinal study, ApoE^-/-^ mice (n=19) infused with Ang II were scanned serially every 7 days for up to 4 weeks **(Supplementary material online, Figure S1A)**.

**Molecular imaging of tropoelastin by *in vivo* MRI**

*In vivo* vessel wall imaging was performed using a 3 Tesla Philips Achieva MR scanner (Philips Healthcare, Best, The Netherlands) equipped with a clinical gradient system (30mT m^–1^, 200mT/m/ms) and a single–loop surface coil (diameter=47mm). Mice were imaged in supine position 30-40min post intravenous injection of the Gd-TESMA (0.2mmol/kg). In the cross-sectional study, a subgroup of animals (n=5 per group) was also scanned 1h (optimal time point) after intravenous injection of Gd-ESMA (0.2mmol/kg) on the previous day for comparison. In the longitudinal study, 10 out of 19 mice where scanned serially only using the Gd-TESMA and the remaining 9 mice were scanned with both the Gd-TESMA and Gd-ESMA contrast agents 24h apart at baseline, 1 and 3 weeks following Ang II-infusion. Gd-ESMA enhancement provide anatomical images of the entire vessel wall and allow normalization of the Gd-TESMA-enhancement to that of vessel wall size **(Supplementary material online, Figure S1A).**

**Mice:** Anesthesia was induced with 5% isoflurane and maintained with 1 - 2% isoflurane during the MRI experiments. Following a 3D GRE scout scan, contrast enhanced angiography images were acquired for visualization of the thoracic and abdominal aorta and the renal branches with a FOV=35×35×16mm, matrix=232×233, in-plane resolution=0.15×0.15mm, slice thickness=0.5mm, repetition time/echo time (TR/TE)=28/6ms, and flip angle=40°. The maximum intensity projection images were used to plan the subsequent late gadolinium enhancement (LGE) MRI and T_1_ mapping scans. A 2D-Look–Locker sequence planned perpendicular to the aorta was used to determine the optimal inversion time (TI) for blood signal nulling. Acquisition parameters were FOV=30×30mm, matrix=76×75, in-plane resolution=0.4×0.4mm, slice thickness=2mm, TR/TE=18/8.3ms, TR between subsequent IR pulses=1000ms, and flip angle=10°. An inversion-recovery 3D fast gradient echo sequence was acquired 30min post-injection and was used for LGE-MRI and visualization of contrast uptake. Imaging parameters were FOV=35×35×12mm, matrix=348×348, in-plane resolution=0.1×0.1×1mm (normal vessel wall thickness=0.2mm), slices=24, TR/TE=27/8.2ms, TR between subsequent infrared pulses=1000ms, and flip angle=30°. T_1_ mapping was performed ~1h post-injection of contrast agent using a 3D Modified-Look-Locker sequence that employs a non-selective inversion pulse with inversion times ranging from 20ms to 2000ms, followed by eight segmented readouts for eight individual images. The two interleaved imaging trains employed to achieve higher temporal resolution result in a set of 16 images per slice with increasing inversion times. For T_1_ mapping, the acquisition parameters were: FOV=36×22×10mm, matrix=180×102, in-plane resolution=0.2×0.21×0.5mm, slices=20, TR/TE=9/4.6ms, and flip angle=10°. Finally, Qflow data were acquired with FOV=40×23mm, matrix=400×228, in-plane resolution=0.1×0.1×2mm, TR/TE=7.2/18ms and flip angle=35°, encoding velocity in the foot-head direction=15cm/s.

***Ex vivo* T_1_ mapping experiments using human aortic aneurysm specimens at 3 Tesla.**

Fresh aneurysmal specimens were imaged before and after soaking in Gd-TESMA **(Supplementary material online, Figure S1B**). **Collection and use of human samples was approved by Guy’s and St Thomas’ Hospital Research Ethics Committee, REC reference #: 13/LO/1685 and written informed consent was obtained from all subjects.** Human AAA specimens (n=4) were obtained fresh from the operating room, washed in phosphate buffered saline, photographed and frozen at −20°C until imaged. On the day of imaging, the specimens were placed in saline and allowed to warm up to room temperature. Each sample was scanned four times: at baseline, after soaking in 1mM of Gd-TESMA for 6h and after rinsing in PBS overnight and after soaking in 1mM of Gd-ESMA for 6h. For the T2W images the imaging parameters were: FOV=30x30x40mm, matrix=64x60, in-plane resolution=0.47x0.47mm, measured slice thickness=2mm, slices=20, TR/TE=2000/30ms, flip angle=90°. For 3D T1 mapping the imaging parameters were: FOV=30x30x40mm, matrix=64x60, in-plane resolution=0.4x0.4mm, measured slice thickness=2mm, slices=20, TR/TE=6/3.1ms, flip angle=35°**.**

**MR image analysis.** Expansion of the aortic aneurysm was assessed by manually segmenting the luminal area as seen on transverse contrast enhanced MRA images using OsiriX (OsiriX Foundation, Geneva, Switzerland). Vascular tropoelastin remodeling was calculated by manually segmenting the visually enhanced region of the vessel wall as seen on the LGE-MRI images after injection of the contrast agent. To ensure that the segmented area encompassed the vessel wall the LGE-MRI images were co-registered and fused with the MRA images. T_1_ values were computed on a pixel-by-pixel basis using a 3 parameter fit implemented in Matlab ^7^. The analysis was performed on anonymized datasets.

**Histological Analysis.**

**Murine.** The suprarenal and the infrarenal aorta, including the renal branches, was removed *en block*, pinned down on a cork and fixed in 10% formaldehyde for 48h (n=4 per time point). Subsequently, the aortas were embedded in paraffin and sectioned transversely (5-μm thick). Masson’s trichrome staining was used to visualise vessel wall morphology and Verhoeff-Van Gieson elastin staining was used to demonstrate normal or pathologic elastin fibres. Immunohistochemistry for tropoelastin was performed using an anti-mouse rabbit polyclonal antibody (1:100, Abcam, ab21600, Cambridge, MA, USA). Digital images were used for computerized planimetry measurements using ImageJ (NIH). Vessel wall area was calculated using the Verhoeff-Van Gieson images as [adventitia area−the luminal area (mm^2^)]. The tropoelastin immunopositive area was segmented on the images and expressed in mm^2^. Internal anatomical markers including the left and right renal arteries, and the last pair of intercostal arteries were used for co-registering the MRI and histological images.

**Human.** After the imaging experiments, the AAAs were fixed in 10% formalin, decalcified, embedded in paraffin, and sectioned transversely at 5μm. Sections were stained for collagen using Masson’s trichrome, elastin with Verhoeff-Van Gieson and tropoelastin by immunohistochemistry (1:100, Abcam, ab21600, Cambridge, MA, USA).

**Western Blotting** was used for quantification of vascular tropoelastin concentration (n=3 per group) as previously described ^1^. The protein concentration of the samples was determined using the RC–DC assay kit (BioRad). All immunoblot measurements were performed using 50mg of protein (equal amounts of total protein loaded per lane was also assured by the GAPDH protein content, used as a loading control).

**Inductively coupled plasma mass spectrometry (ICP–MS).** ICP-MS was used for quantification of gadolinium (Gd^3+^) concentration in the vessel wall (n=4 per group) as previously described ^1^.

**Elisa:** Soluble levels of tropoelastin in serum were quantified using the mouse Tropoelastin (TE) ELISA (Kit MBS9356848) (n=6/ group).

**Statistical analysis.** A Shapiro-Wilk t test (p>0.05) and visual inspection of histograms, Q-Q plots and box plots were used to test for normal distribution of the variables. A Levene’s test was used to test for equality of variances in the samples (homogeneity of variances) (p > 0.05). Two-group comparison of continuous variables was performed with a non-parametric Mann-Whitney test (e.g., histological tropoelastin area in control and dilated samples, MRA and LGE-MRI area with Gd-TESMA in non-treated vs statin/aspirin-treated animals, Gd-ESMA and Gd-TESMA LGE area over time of Ang II-infusion) or a Wilcoxon signed rank test for paired samples (e.g., LGE-MRI after Gd-ESMA and Gd-TESMA injection in the same animal or human tissue). Multiple group comparisons of non-normally distributed data were performed with a Kruskal-Wallis nonparametric ANOVA test and a Dunn’s post-hoc test (e.g., LGE-MRI and R_1_ with Gd-TESMA in control vs non-dilated vs dilated aortas, ICP-MS, Western blotting, LGE-MRI Gd-TESMA volume in small–medium-large dilation, histological tropoelastin area in small-medium-large aortic dilation, Gd-ESMA and Gd-TESMA LGE area over time of Ang II-infusion). Correlation analysis was performed with a Spearman test. Linear regression analysis was used to determine the relation between the deposition of tropoelastin and aortic expansion. Aortic dilation were classified in small (5.44-11.90mm^3^), medium (11.91-40.58mm^3^), and large (>40.59-105 mm^3^). Cut-off values were selected such that an equal number of cases were included in each category. The interobserver variability was assessed by using the interclass correlation coefficient (ICC) for continuous variables.

The box-whisker plots show the mean values, interquartile range, first and third quartiles and the whiskers represent the minimum to maximum values. GraphPad Prism 5.0 (GraphPad Software, Inc., La Jolla, California, USA) was used for the statistical analysis. p-values < 0.05 were used to define statistical significance.

**Supplementary material online**

**Figure S1**


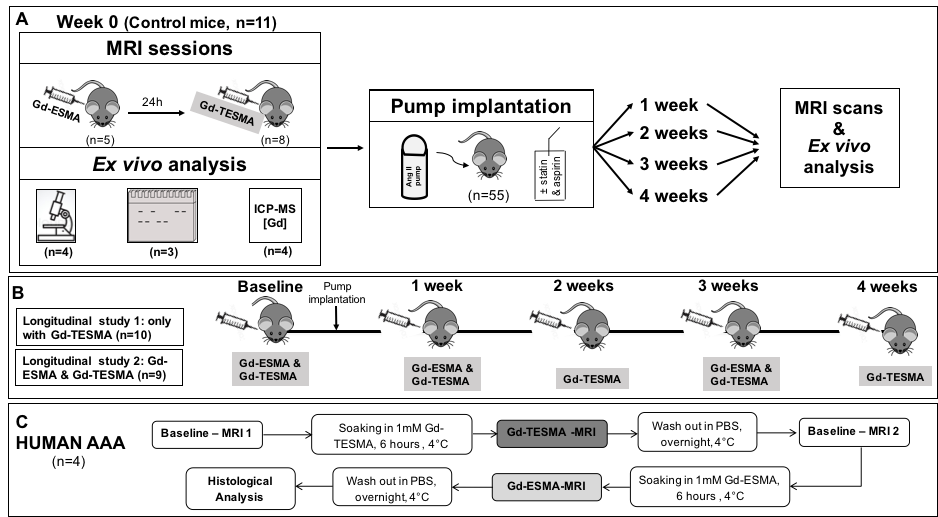


**Figure 1: (A) Cross-sectional (n=66) and (B) longitudinal (n=19) timeline for *in vivo* imaging of matrix remodeling in abdominal aortic dilation using an Ang II-infused ApoE^-/-^ murine model at 3 Tesla and (C) *ex vivo* imaging of matrix remodeling in excised human aortic aneurysm tissues (n=4) at 3 Tesla.**

**Figure S2**

**Figure 2: MR imaging of aortic dilation in an angiotensin II-infused ApoE^-/-^ murine model at 3T. (A-E)** 3D reformatted *in vivo* MR angiograms show the formation of dilated aortic segments of varying sizes at different time points after implantation of the pumps and infusion of Ang II. **(F-G)** A representative *ex vivo* preparation shows a suprarenal dilation in an animal infused with Ang II for 1 week. **Table 1:** Summary of Ang II-induced aortic aneurysms and dissections in ApoE^-/-^ mice. Infusion of Ang II induced aortic dilation in the suprarenal aorta in 65.5% of mice, as previously described ^4, 5^. The frequencies of aortic aneurysm and dissections were similar in this cohort of mice. Ao: aorta; RRA: right renal artery; LRA: left renal artery, CA: celiac artery.

**Figure S3**

**Figure 3: Comparison of the aortic wall enhancement using Gd-ESMA (total elastin) and Gd-TESMA (tropoelastin) during Ang II infusion shows that the uptake of Gd-TESMA was confined within the dilated aorta.** In our study, we compared the vessel wall uptake after administration of Gd-ESMA and Gd-TESMA in a sub-group of animals (n=5 per time point), imaged 24h apart, after infusion of Ang II. We found that although both agents enhance the aortic wall, the ability of Gd-ESMA to detect elastin remodeling during Ang II infusion was revealed only when the MRI slices were separated into those containing dilated aortic segments and those that did not. Conversely, accumulation of Gd-TESMA was confined only within dilated aortic walls at all time points of Ang II infusion. **(A)** Quantitatively, the LGE-MRI area measured after Gd-ESMA administration at different time points of Ang II infusion did not show any significant differences over time. **(B)** Conversely, when using the Gd-TESMA there was a significant increase in the aortic wall enhancement with aortic dilation and duration of Ang II infusion. **(C)** However, the ability of Gd-ESMA to detect changes in elastin deposition during aortic dilation and progression was revealed only when the slices were separated into those containing dilated aortas and those that did not. When this analysis was performed there was a significant increase in LGE-MRI area within dilated vessel wall compared to non-dilated wall and also compared to control vessel walls at most time points of Ang II infusion suggesting a net increase in elastin/tropoelastin deposition. **(D)** Accumulation of Gd-TESMA was confined within the dilated wall at all time points of Ang II infusion. Quantitative data were compared with a Kruskal-Wallis ANOVA followed by a Dunn’s post-hoc test (A,B) and a Mann-Whitney test (C,D). A, C: n=5/ time point and B, D: n=8/ time point.

**Figure S4**

**Figure 4: Correlations between *in vivo* MRI and *ex vivo* histology.**

**(A-B-C)** *In vivo* tropoelastin remodeling as measured by late gadolinium enhancement (LGE) MRI and T_1_ mapping after the administration of Gd-TESMA correlates with the aortic cross-sectional area and diameter. **(D)** The *ex vivo* tropoelastin content measured by immunohistochemistry correlates with the size of the histological aortic cross-sectional area. **(E)** The *ex vivo* immunohistological tropoelastin content correlates with the late gadolinium enhanced area as measure by MRI after administration of Gd-TESMA. Interclass correlation coefficient (ICC) analysis showed a very good inter-reader agreement for the analysis of the MRI and histological data. The ICCs for measuring the MRA cross-sectional data, the LGE-MRI and tropoelastin immunohistochemistry were 0.97 (95% CI 0.94-0.99), 0.92 (95% CI 0.84-0.96), and 0.93 (95% CI 0.77-0.98), respectively.

**Figure S5**

**Figure 5: Measurement of soluble tropoelastin in serum.** Quantification of soluble tropoelastin in serum showed no differences between control (n=6), Ang II-infused mice with dilated aortas (n=6), and Ang II-infused mice treated with pravastatin and aspirin (n=6).

**References**

1. Phinikaridou A, Lacerda S, Lavin B, Andia ME, Smith A, Saha P, Botnar RM. Tropoelastin: A novel marker for plaque progression and instability. Circ Cardiovasc Imaging 2018;**11**(8).

2. Manning MW, Cassi LA, Huang J, Szilvassy SJ, Daugherty A. Abdominal aortic aneurysms: fresh insights from a novel animal model of the disease. Vasc Med 2002;**7**(1):45-54.

3. Daugherty A, Cassis LA, Lu H. Complex pathologies of angiotensin II-induced abdominal aortic aneurysms. J Zhejiang Univ Sci B 2011;**12**(8):624-8.

4. Daugherty A, Manning MW, Cassis LA. Angiotensin II promotes atherosclerotic lesions and aneurysms in apolipoprotein E-deficient mice. J Clin Invest 2000;**105**(11):1605-12.

5. Saraff K, Babamusta F, Cassis LA, Daugherty A. Aortic dissection precedes formation of aneurysms and atherosclerosis in angiotensin II-infused, apolipoprotein E-deficient mice. Arterioscler Thromb Vasc Biol 2003;**23**(9):1621-6.

6. Daugherty A, Manning MW, Cassis LA. Antagonism of AT2 receptors augments angiotensin II-induced abdominal aortic aneurysms and atherosclerosis. Br J Pharmacol 2001;**134**(4):865-70.

7. Makowski MR, Wiethoff AJ, Blume U, Cuello F, Warley A, Jansen CH, Nagel E, Razavi R, Onthank DC, Cesati RR, Marber MS, Schaeffter T, Smith A, Robinson SP, Botnar RM. Assessment of atherosclerotic plaque burden with an elastin-specific magnetic resonance contrast agent. Nat Med 2011;**17**(3):383-8.
